# Comment on: Estimating Economic Burden of Cancer Deaths Attributable to Smoking in Iran in 2012

**Published:** 2016-06-14

**Authors:** Sara Geravandi, Mohsen Pakdaman

**Affiliations:** ^a^ Department of Health Economics, School of Public Health, Tehran University of Medical Sciences, Tehran, Iran; ^b^ Department of Health Care Management, School of Public Health, Shahid Sadoughi University of Medical Sciences, Yazd, Iran.

## Dear Editor-in-Chief

In November 2015, an original article, entitled, ‘‘Estimating Economic Burden of Cancer Deaths Attributable to Smoking in Iran in 2012’’ by Rezaei et al. was published in Journal J Res Health Sci^1^.We would like to propound a few points that seem to be complementary‏ in the use of the methodology in this article.

First, although the study aimed to estimate the economic burden of cancer-attributable deaths due to smoking, but only indirect cost was calculated. It is important that direct costs of disease containing direct medical and nonmedical costs account for a considerable proportion of the total costs across countries especially in low- and middle-income countries^2^. Ignoring the direct costs, the economic burden of disease tends to be underestimated. Anyway, in some high-income countries the indirect cost can be the major part of the total cost. Such differences could be explained by variation across labor markets. There is much higher average earning in the Western market economics^3^.

Second, the human capital approach (HCA) was used to measure indirect costs. It estimates production losses by calculating expected earnings of work productivity foregone due to morbidity and mortality. HCA has some disadvantages: working-age, higher earning potential are valued over the old, young, men, educated, assumes earnings reflect productivity, excludes social impact of productivity loss. However, the main concern with this approach is that HCA accounts every hour the patient is absent as one hour of lost production. In the other words, the traditional human capital approach measures potential productivity loss. But a worker who is absent due to mortality or long term disability is replaced by new worker. Infact HCA measures the productivity losses from the patient perspective. Due to the fact that an estimate of lost production using the HCA tends to be overestimated, the friction cost approach (FCA) is usually applied. FCA offers insight on the need to consider how lost productivity occurs. If a cancer patient absent from work due to mortality or disability, new or existing workers make up for the vacant job by the employer.

The friction cost method estimates the productivity costs by calculating the value of production losses only during the friction period (time between start of absences of work and replacement)estimated to be about 90 days. FCA adjusts productivity loss by multiplying the results of the HCA by the friction period and then dividing this product by the average duration of work incapacity period (which must be calculated for patients with cancer)-^6^.

**Figure 1:**
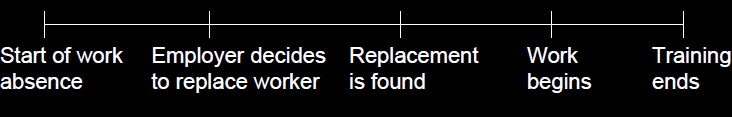


## Friction cost time line

Another comment in reference to the article is that no sensitivity analysis to test the robustness of the results was performed. Sensitivity analysis is recommended anytime there is uncertainty. Cost-of-illness studies rely on estimations with unstable degrees of uncertainty. Therefore, it is essential to differ in the assumptions, in order to determine the ranges of possible values the costs can take^7, 8^.

Lastly, because of data restrictions, the analysis relied on data on the relative risks of smoking were obtained from the study conducted in Korea, and the prevalence of various status of smoking from the study conducted in 2005. Therefore, the study implies that further research should be undertaken to discover more recent data as for Iranian population. Addressing such limitations in the future studies, more accurate and comparable estimation of costs can be achieved.

At the end, the authors should be appreciated for their preceding attempt to estimate the economic burden of major cancer due to smoking in Iran.

Sara Geravandi (MSc)^a^, Mohsen Pakdaman (PhD)^b^*

^a^ Department of Health Economics, School of Public Health, Tehran University of Medical Sciences, Tehran, Iran

^b^ Department of Health Care Management, School of Public Health, Shahid Sadoughi University of Medical Sciences, Yazd, Iran.

*****Correspondence to:***** *Mohsen Pakdaman (PhD)*

**E-mail:** *mohsen66pakdaman@gmail.com*

## Reply

There are three comments as follows:

Firstly, why we did not include other costs such as health cost in the study. The aim of this study was to examine the economic burden of deaths due to major cancer in Iran for 2012 attributable to smoking. The aim of the study was only the costs associated with death due to cancers and not after the death. This is consistence with other studies^1-4^. There are two popular approaches to estimate the indirect cost such as HCA and willingness to pay (WTP)^5-7^. In most of studies, the HCA was used and the evidence about WTP rarely documented. In the literature review, we did not find any studies using FCA to estimate the indirect costs. We mentioned the limitation of HCA in the study at the end of discussion section. The study was not conducted the sensitivity analysis which is one of the limitations of the study.

Satar Rezaei (MSc)^a^, Ali Akbari Sari (PhD)^b^*

^a^ Department of Health Management and Economics, School of Public Health, Tehran University of Medical Sciences, Tehran, Iran

^b^ Department of Global Health and Public Policy, School of Public Health, Tehran University of Medical Sciences, Tehran, Iran

*****Correspondence to:***** *Ali Akbari Sari (PhD)*

**E-mail:** *akbarisari@tums.ac.ir*

## References

[1] Rezaei S, Akbari Sari A, Arab M, Majdzadeh R, Mohammadpoorasl A (2015). Estimating Economic Burden of Cancer Deaths Attributable to Smoking in Iran in 2012. J Res Health Sci.

[2] Oh IH, Yoon SJ, Yoon TY, Choi JM, Choe BK, Kim EJ (2012). Health and economic burden of major cancers due to smoking in Korea. Asian Pac J Cancer Prev.

[3] Luengo-Fernandez R, Leal J, Gray A, Sullivan R (2013). Economic burden of cancer across the European Union: a population-based cost analysis. Lancet Oncol.

[4] Koopmanschap MA, Rutten FF, van Ineveld BM, van Roijen L (1995). The friction cost method for measuring indirect costs of disease. J Health Econ.

[5] Johns H. Creative Commons Attribution-NonCommercial-Share Alike License. Copyright 2007. The Johns Hopkins University and Kevin Frick.

[6] Koopmanschap MA, van Ineveld BM (1992). Towards a new approach for estimating indirect costs of disease. Soc Sci Med.

[7] Ebadifard Azar F, Rezapour A. Health Care Economics. 2^nd^ ed. Tehran: Ebadifar Pub; 2014.

[8] Claxton K, Schulpher M, McCabe C, Briggs A, Akehurst R, Buxton M, Brazier J, O’Hagan A (2005). Probabilistic sensitivity analysis for NICE technology assessment: not an optional extra. Health Economics.

[9] Khorasani S, Rezaei S, Rashidian H, Daroudi R (2015). Years of potential life lost and productivity costs due to premature cancer-related mortality in Iran. Asian Pac J Cancer Prev.

[10] Hanly PA, Sharp L (2014). The cost of lost productivity due to premature cancer-related mortality: an economic measure of the cancer burden. BMC Cancer.

[11] Hanly P, Soerjomataram I, Sharp L (2015). Measuring the societal burden of cancer: The cost of lost productivity due to premature cancer‐related mortality in Europe. Int J Cancer.

[12] Yabroff KR, Bradley CJ, Mariotto AB, Brown ML, Feuer EJ (2008). Estimates and projections of value of life lost from cancer deaths in the United States. J Natl Cancer Inst.

[13] Yabroff KR, Lund J, Kepka D, Mariotto A (2011). Economic burden of cancer in the United States: estimates, projections, and future research. Cancer Epidemiol Biomarkers Prev.

[14] Daroudi R, Sari AA, Nahvijou A, Kalaghchi B, Najafi M, Zendehdel K (2015). The economic burden of breast cancer in Iran. Iran J Public Health.

[15] Max W, Rice DP, Sung H-Y, Michel M. Valuing human life: estimating the present value of lifetime earnings, 2000. San Francisco: Center for Tobacco Control Research and Education; 2004.

